# Recent Progress on Molecular Photoacoustic Imaging with Carbon-Based Nanocomposites

**DOI:** 10.3390/ma14195643

**Published:** 2021-09-28

**Authors:** Songah Jeong, Su Woong Yoo, Hea Ji Kim, Jieun Park, Ji Woo Kim, Changho Lee, Hyungwoo Kim

**Affiliations:** 1 School of Polymer Science and Engineering, Chonnam National University, 77 Yongbong-ro, Buk-gu, Gwangju 61186, Korea; songa1229@naver.com (S.J.); hi335@naver.com (H.J.K.); jieunp11@naver.com (J.P.); bigrnjs7@gmail.com (J.W.K.); 2 Department of Nuclear Medicine, Chonnam National University Hwasun Hospital, 264, Seoyang-ro, Hwasun-eup, Hwasun-gun 58128, Jeollanam-do, Korea; yoosw.md@gmail.com; 3 Department of Nuclear Medicine, Chonnam National University Medical School, 160, Baekseo-ro, Dong-gu, Gwangju 61469, Korea; 4 Department of Artificial Intelligence Convergence, Chonnam National University, 77 Yongbong-ro, Buk-gu, Gwangju 61186, Korea

**Keywords:** photoacoustic, carbon materials, molecular imaging, contrast agents, theragnosis

## Abstract

For biomedical imaging, the interest in noninvasive imaging methods is ever increasing. Among many modalities, photoacoustic imaging (PAI), which is a combination of optical and ultrasound imaging techniques, has received attention because of its unique advantages such as high spatial resolution, deep penetration, and safety. Incorporation of exogenous imaging agents further amplifies the effective value of PAI, since they can deliver other specified functions in addition to imaging. For these agents, carbon-based materials can show a large specific surface area and interesting optoelectronic properties, which increase their effectiveness and have proved their potential in providing a theragnostic platform (diagnosis + therapy) that is essential for clinical use. In this review, we introduce the current state of the PAI modality, address recent progress on PAI imaging that takes advantage of carbon-based agents, and offer a future perspective on advanced PAI systems using carbon-based agents.

## 1. Introduction

In recent decades, molecular imaging has become an essential technology thanks to advances in imaging techniques and the development of novel nanocomposites. It has been widely applied in areas ranging from basic biomedical research, for example, the monitoring of cell structure and functionality [1,2,3] through clinical implementations such as early cancer diagnosis and therapy monitoring, and, when partnered with nuclear medicine and CT-imaging, observing vessel angiogenesis [4,5,6,7]. In particular, optical imaging techniques such as confocal microscopy (CM), multiphoton microscopy (MPM), and fluorescence imaging (FLI) have accelerated the rapid development of molecular imaging based on the relatively safe ultra-sensitive interactions between radiation and target nanomaterials [8,9,10]. Although these optical methods have the benefits of high spatial resolution (1–10 µm) and spectroscopic differentiation, they suffer from the significant reduction of light intensity with depth, resulting from scattering and absorption by biological tissues. Normally, the imaging depth of pure optical imaging is theoretically limited to 1 mm and computational diffused imaging methods cannot maintain their high resolution in deep tissue [11,12,13,14].

**Figure 1:**
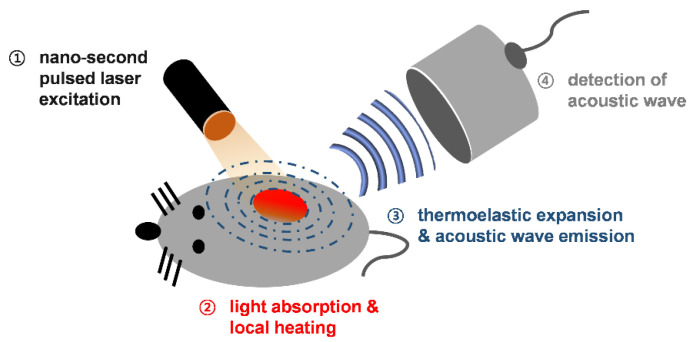
Schematic of the basic principles of photoacoustic imaging.

Photoacoustic imaging (PAI) is a new biomedical technology that provides dual image contrast by merging optical and acoustic imaging techniques. Thanks to its hybrid imaging properties, PAI is able to visualize relatively deep tissue (i.e., several cm) while maintaining the excellent resolution of its acoustic component [15,16,17,18]. Figure 1 indicates the basic principles of PAI. When a high-energy laser pulse strikes the sample, the light pulse is absorbed and converted into thermal energy. As the sample’s temperature increases, thermoelastic expansion occurs accompanying acoustic emission; this phenomenon is called the photoacoustic (PA) effect [19]. The broadband acoustic wave emitted is then detected by ultrasound sensors. Because there is less scattering of an acoustic wave from living tissues compared to incident light, PAI achieves relatively deep tissue imaging compared to pure optical imaging. Given its ability to capture endogenous contrast absorbers in the body with an optimal wavelength laser, PAI can provide physical mapping information on blood vessels, lipids, collagen, and melanin [20,21,22,23,24], as well as estimates of physiological variables such as hemoglobin concentration, saturated oxygen ratio, and blood flow [25,26,27,28]. These advantages of PAI can contribute to resolving many problems in fundamental science and pre- and clinical research fields [29,30,31,32,33].

The endogenous PA contrasts in the living body help to achieve diverse contrast in molecular PA imaging of individual organs and biological components, but the insensitivity to low concentrations of biomolecules and their absorption of visible light limits the ability to provide strong PA signals from deep tissues [34]. In addition, white and transparent organs, such as the intestines, lymph nodes, and bladder cannot produce sufficient PA signals because of their extremely low light absorption. Therefore, there has been an ongoing need for exogenous PA contrast agents to achieve high PA sensitivity, deep tissue imaging, and specific targeting of non-pigmented organs [35,36,37,38,39,40]. A variety of materials have been used as the exogenous contrast agent, including small-molecule chromophores, π-conjugated polymer-type organic semiconductors, gold nanoparticles, or organic-inorganic hybrid materials, and they have shown the PA imaging capability along with desired properties such as biotic degradation, biodistribution, or renal clearance as summarized before [41]. Meanwhile, carbon-based nanomaterials have attracted attention owing to their unique shape, extended π-conjugation, synthetic flexibility for surface engineering, large surface area, or interesting photophysical properties, which are of the main focus of this review paper as shown below.

Carbon nanomaterials have been rapidly developed over the past decades and have received great attention from not only academia but also industry. The sp^2^ nanomaterials have been designed into different dimensions such as 0-dimensional nanodots, 1-dimensional nanotubes, 2-dimensional nanosheets, and more complex structures such as nanohorns and nano-onions, which then have application in many fields such as energy, electronics, the environment, and biomedicine [42]. In general, nanomaterials can have large specific surface areas and enhanced optoelectric properties, which bring about the formulation of affordable nanoprobes for bio-imaging; further, they can provide a theragnostic platform, i.e., a combination of diagnosis and therapy. In all bio-related applications, the cytotoxicity of materials is of great importance. In view of this, nanotubes with high aspect ratios are controversial because their pathogenicity is similar to asbestos [43]. For example, some multiwalled carbon nanotubes such as MWCNT-7 have been recently classified as a potential carcinogen by the International Agency for Research on Cancer (IARC) and restricted in the EU, while others remain not classifiable [44]. Physicochemical parameters such as diameter, stiffness, or biopersistence of the materials are considered as major drivers for their biological reactivity, which leaves room for tailoring the nanotubes to reduce nanotoxicology [45]. Additionally, thorough purification or surface modification can significantly alleviate the negative effects [46,47]. Nonetheless, special caution should be paid to using long and rigid carbon nanotubes. Other carbon materials are non-cytotoxic or show only low acute toxicity so far, which reveals their promise. Here, we classify these carbon materials in accordance with their overall shapes and briefly summarize the recent relevant research.

This review paper focuses on recent developments of photoacoustic methodologies and carbon-based imaging agents that offer the enhancement of optical contrast and further provide therapeutic effects. Considering that the carbon-based agents have been seldom summarized before, we anticipate that this review paper will, particularly, help readers who follow the recent progress on photoacoustic bio-imaging fields in which carbon materials are used as a functional imaging agent.

## 2. Multiscale Photoacoustic Imaging Systems

PAI systems can be classified as photoacoustic microscopy (PAM) or photoacoustic computed tomography (PACT), according to the system capabilities (i.e., spatial resolution, imaging depth) and the hardware configuration (e.g., single- or multi-acoustic transducers) [48,49]. 

### 2.1. Photoacoustic Microscopy (PAM)

**Figure 2:**
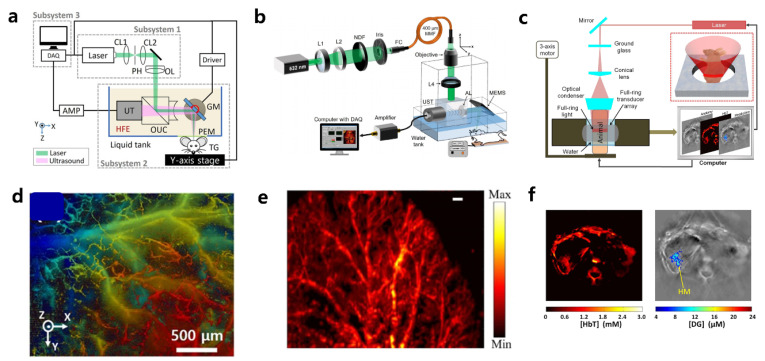
Multiscale PAI systems. (**a**) Schematic of an OR-PAM system using a high-speed galvanometer scanner. Resolution is determined by the focused laser beam spot size. (**b**) Schematic of an AR-PAM system. Resolution is determined by the ultrasound detection system. (**c**) Schematic of a ring-shaped whole-body PACT system. (**d**) Microvasculature of a mouse ear obtained using OR-PAM. (**e**) Vascular imaging using AR-PAM at an ultrasound frequency of 75 MHz. (**f**) PACT images of Hbt concentration (left) and a kidney tumor-specific contrast agent (in color) fused with a structure image (right, in grayscale). Reproduced with permission from [50,51,52]. Copyright, Nature Publishing Group (2016) [50], John Wiley and Sons (2016) [51], SPIE (2012) [52].

PAM can create high-resolution images using a focused laser beam or a focused ultrasound capture scheme. Thanks to the high speed of lasers and fast scanning techniques such as micro-electro-mechanical systems (MEMS) and galvo-scanners, PAM can produce images in real-time [50,51,52,53,54,55,56,57]. When the spatial resolution is determined by a small, focused laser beam spot, the technique is called optical-resolution PAM (OR-PAM) (Figure 2a). OR-PAM can show the microvasculature of a mouse ear with a resolution on the order of several micrometers (Figure 2d). However, the penetration depth of OR-PAM is normally limited to approximately 1 mm because of the light diffusion in biological tissue. When the tighter ultrasonic detection bounds become the determining factor for spatial resolution, it is called acoustic-resolution PAM (AR-PAM) [51,58,59,60]. Even when AR-PAM does not provide better spatial resolution than OR-PAM, it can achieve a greater penetrating depth of a few millimeters because there is less ultrasound scattering in biological tissue (Figure 2b). Optical excitation is realized through dark-field illumination in AR-PAM, achieving a spatial resolution of 53 μm with an imaging depth of 1.8 mm in vivo (Figure 2e).

### 2.2. Photoacoustic Computer Tomography (PACT)

PACT acquires 2- and 3-dimensional PA images with multi-array ultrasound transducers and CT reconstruction methods [52,61,62,63,64]. Multifold ultrasound signal detection with single laser irradiation contributes to achieving real-time PAI with improved image acquisition speed without mechanical scanning approaches (Figure 2c). Depending on the center frequency and the geometry of the ultrasound elements of the probe, PACT can provide an imaging depth of several centimeters. By integrating the multi-dimensional PA data acquired from the multi-array transducer, it is possible to obtain enough information to reconstruct a volumetric PA image of the original feature. Depending on the ultrasound detector type (e.g., arch, ring) and data acquisition continuations, diverse optimized PACT reconstruction algorithms have been proposed. PACT can be implemented for macroscopic imaging, and it has been successfully used for various types of structural, functional, and molecular imaging. In combination with a tissue-specific contrast probe, targeted PACT imaging can be achieved (Figure 2f). Although real-time imaging can be achieved using PACT, it is subject to distortion from artifacts in the image reconstruction process.

## 3. Carbon Materials for Photoacoustic Imaging

### 3.1. Carbon Nanotubes

**Figure 3:**
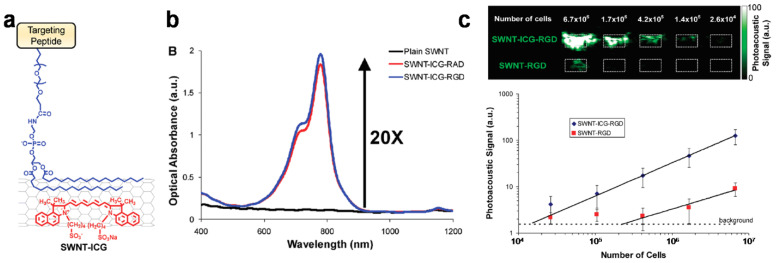
(**a**) Illustration of SWNT conjugated with ICG dye (SWNT-ICG). (**b**) Optical spectra of plain SWNT (black), SWNT-ICG-RGD (blue), and SWNT-ICG-RAD (red). (**c**) Photoacoustic image (upper) and quantitative photoacoustic signals (lower) from cancer cells exposed to SWNT-ICG-RGD and SWNT-RGD. Reprinted with permission from [70]. Copyright, American Chemical Society (2010).

The carbon nanotube is theoretically made from a graphene sheet rolled up into a seamless cylinder [65]. The carbon nanotube was first introduced in 1991 [66] and many studies in the last few years have shown promising applications in the fields of medicine and nanotechnology because of their interesting functionalities. Kim et al. made gold nanotubes (GNT), which are essentially carbon nanotubes coated with a thin gold layer [67]. By conjugation of the lymphatic endothelial receptor antibody, GNTs were used to target specific lymphatic vessels by PAI and for photothermal therapeutic applications. The functional composite materials showed enhanced contrast in lymphatic vessels under NIR irradiation, which could result in more efficient PA imaging and photothermal therapy and had biocompatibility with minimal toxicity. Furthermore, following bio-conjugation with the antibody (anti-LYVE-1) that is specific to the lymphatic endothelial hyaluronan receptor-1 (LYVE-1), the resultant materials allowed mapping of the lymphatic endothelial cells (LECs); this may lead to a useful alternative to the current fluorescent labelling method that is limited in its in vivo applications because of potential cytotoxicity, immune response, photobleaching, and signal interference [68]. De la Zerda et al. conjugated the single-walled carbon nanotube (SWNT) with Arg-Gly-Asp (RGD) peptide for tumor-targeting PAI agents [69]. SWNT-RGD showed a significantly higher PA signal in the mouse tumor than plain SWNT in ex vivo specimens. The in vivo imaging in the mouse tumor also showed a higher PA signal with SWNT-RGD than with plain SWNT. To enhance the PA signal, they attached Indocyanine Green (ICG) dye to the SWNT-RGD (Figure 3a) [70]. SWNT-ICG-RGD particles showed a 20-fold higher absorbance than plain SWNTs (Figure 3b). As a result of its high PA signal, the SWNT-ICG-RGD achieves better sensitivity for detecting tumor cells; the process detects a 20-times lower number of tumor cells than with previous SWNT-RGD particles (Figure 3c). Therefore, by combining with other agents, the carbon nanotube can be used to create advanced PAI agents with enhanced sensitivity and specificity for biomedical applications.

### 3.2. Carbon Nanohorns

**Figure 4:**
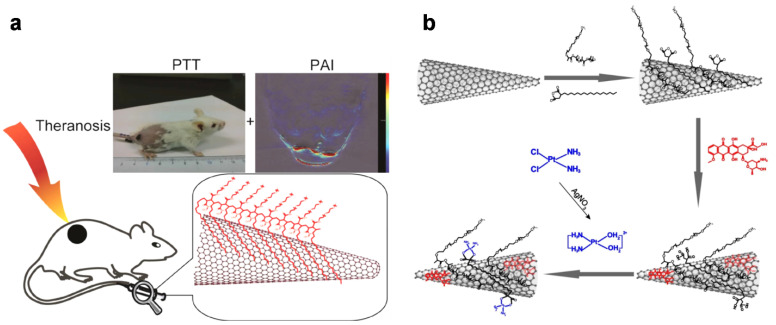
(**a**) Scheme to illustrate near-infrared photothermal therapy and photoacoustic imaging with SWNHs/C_18_ PMH-PEG. Reproduced with permission from [74]. Copyright, John Wiley and Sons (2014). (**b**) Schematic illustration of the preparation of dual drug-loaded SWNHs. Reproduced with permission from [75]. Copyright, Ivyspring International Publisher (2018).

Single-walled carbon nanohorns (SWNHs) are from the family of nanocarbons and are, in principle, obtained from graphene sheets, similar to single-walled carbon nanotubes (SWCNTs), by oblique rolling into a cone shape. The conical-shaped material is sp^2^-hybridized and semi-conducting, similar to CNTs, and typically forms dahlia-like or bud-like aggregates, which are composed of thousands of horns. The overall diameter of SWNH aggregates measure from 80–100 nm on average, which is favorable for the enhanced permeability and retention (EPR) effect. Furthermore, the aggregates are biocompatible and free from metal impurities and absorb long-wavelength light (e.g., red or near-infrared). Therefore, SWNHs have been widely used in biomedical applications, including drug delivery and bio-imaging [71,72,73]. Figure 4 shows the role of SWCNTs in PA imaging applications. Notably, Chen et al. encapsulated SWCNTs with a hydrophilic, polyethylene glycol-based polymer (C_18_PMH-PEG) to maintain water dispersibility and then investigated the imaging capability (Figure 4a). The composite materials absorbed NIR light, showing their potential in photothermal therapy as well as in PA imaging in vivo [74]. Figure 4b presents multifunctional SWNHs that enable synergistic therapeutic effects. Yang et al. coated SWNHs with biocompatible polymers {i.e., poly (maleic anhydride-alt-1-octadecene) and poly (ethylene glycol) methyl ether-*b*-poly (_D_,_L_-lactide)}, and loaded two types of drugs such as cisplatin and doxorubicin. The drug-loaded SWNHs not only generated a PA signal and heat when irradiated at 808 nm, but also exhibited a sustained release of drugs in tumors, which enabled PA imaging-guided chemo-photothermal therapeutics [75].

### 3.3. Carbon Nanodots

**Figure 5:**
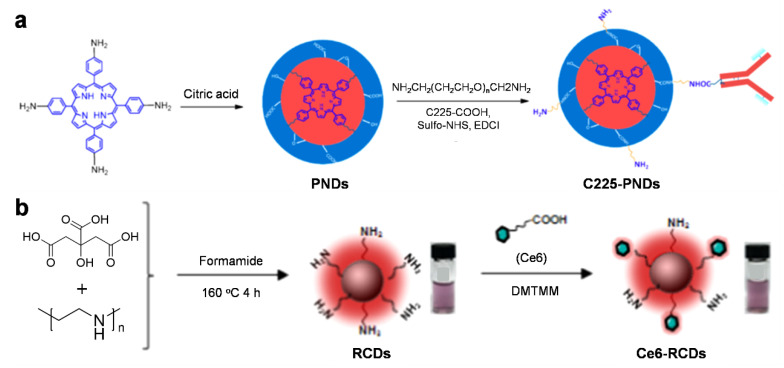
(**a**) Proposed formation pathway of PNDs and the synthesis routes of C225-PNDs. Reprinted with permission from [77]. Copyright, American Chemical Society (2018). (**b**) Schematic of the preparation of amino-rich red emissive carbon dots (RCDs) and Ce6-modified RCDs (Ce6-RCDs). Reprinted with permission from [78]. Copyright, American Chemical Society (2019).

Carbon nanodots (C-dots) are a relatively new material among nanocarbons; their diameter is typically less than 10 nm. Interestingly, these nanoscale carbon materials exhibit unique luminescent properties as a result of quantum confinement effects, similar to metal-based semiconducting quantum dots (QDs). In contrast to other nanocarbons, they have good water dispersibility; furthermore, size tunability, ease of synthesis, chemical inertness, and biocompatibility have attracted great attention and broadened the range of potential applications in the bioimaging field [76]. For the formation, small molecules or polymers are widely used to form and further functionalize the carbon matrices. Recently, Wu et al. prepared functional porphyrin-incorporated carbon nanodots (PNDs) based on citric acids via a solvothermal reaction. The implantation of porphyrin units significantly enhanced the light absorption in the NIR region, increasing penetration depth for in vivo photoacoustic imaging and also enabling photodynamic therapy (PDT). Furthermore, the targeting moiety Cetuximab (C225), which recognizes the epidermal growth factor receptor (EGFR), was introduced onto the outer surface of particles to enable the imaging and therapeutic modalities (C225-PNDs). Thus, a precise and efficient therapy could be performed for cancer cells such as HCC827 and MDA-MB-231 cells using excitation at 808 nm through a two-photon absorption process (Figure 5a) [77]. Sun et al. demonstrated multifunctional C-dot-based nanomaterials that are capable of multimodal imaging (i.e., fluorescence and PA imaging) and useful for photothermal and photodynamic therapies. The combination of citric acid and polyethyleneimine (PEI) provided amine-rich red emissive carbon dots (RCDs) after a solvothermal process. The exposed amine groups on RCDs were further modified with the photosensitizer chlorin e6 (Ce6), leading to the formation of Ce6-RCDs, which helps generate singlet oxygen (^1^O_2_) upon irradiation with a NIR laser. These materials, therefore, show a high efficacy for cancer therapy in vitro and in vivo, even with a reduced laser power density (e.g., 0.5 W cm^−2^ at 671 nm) (Figure 5b) [78].

**Figure 6:**
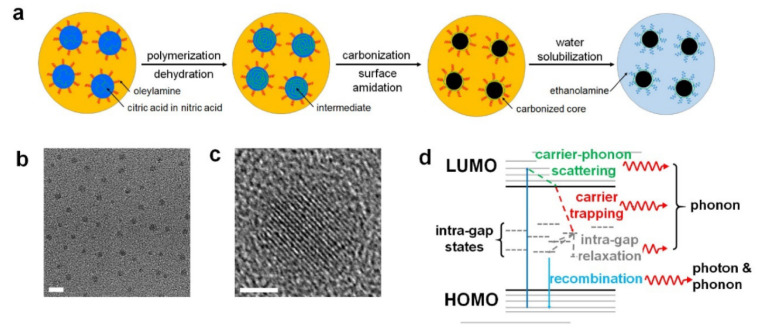
Synthesis of N-CNDs and the effect of nitrogen doping. (**a**) Synthesis of N-CNDs. (**b**) TEM image of N-CNDs (scale bar = 5 nm). (**c**) Partial graphitic structures in the core of N-CNDs (scale bar = 2 nm). (**d**) Illustration of electronic structures and nitrogen-induced intra-gap states of N-CNDs. Reproduced with permission from [39]. Copyright, Ivyspring International Publisher (2016).

Biodegradability and renal clearance are of great importance for clinical use, given that exogenous chemical agents can easily accumulate in organs such as the liver without being excreted [16]. Figure 6 shows C-dot imaging agents that degrade under physiological conditions and, therefore, possess enhanced biocompatibility permitting practical biomedical applications, although some carbon-based agents have undergone considerable chemical and physical changes and unavoidably resulted in cytotoxicity [79]. Lee et al. reported nitrogen-doped C-dots (NCNDs) that have strong absorption in the near-infrared region, high photo-stability, and excellent biodegradability. Therefore, biodegradable dots can not only provide deep-tissue PA imaging but also perform photo-thermal therapy for tumors. The materials showed biodegradation as designed, and renal clearance was confirmed after degradation [39].

**Figure 7:**
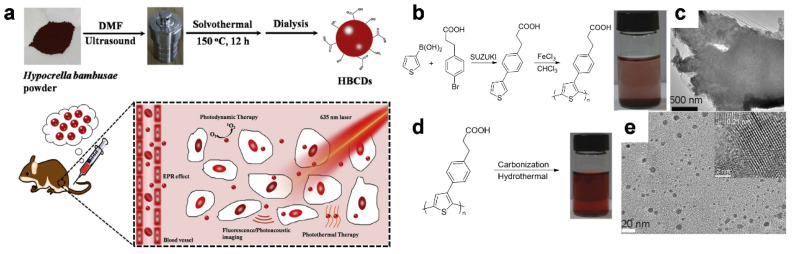
(**a**) Schematic illustration of HBCDs derived from *Hypocrella bambusae* (HB) for bimodal FL/PA imaging and synergistic PDT/PTT of cancer. Reprinted with permission from [81]. Copyright, Elsevier (2014). (**b**–**e**) Preparation and characterization of C-dots: (**b**) synthetic route to PPA (inset: the photograph shows that the PPA is insoluble in water). (**c**) TEM image of PPA. (**d**) synthetic route to C-dots (inset: the photograph shows water-dispersible C-dots). (**e**) TEM and high-resolution TEM (HRTEM) images of C-dots. Reproduced with permission from [82]. Copyright, John Wiley and Sons (2015).

Notwithstanding small molecules, including citric acid (CA), that have been used for the preparation of C-dots, many polymeric materials such as naturally occurring substances or synthetic polymers have also been used as a precursor [80]. Jia et al. used *Hypocrella bambusae* (HB), a parasitic fungus in bamboo, to synthesize C-dots via a solvothermal method without the need for any additives [81]. HB, used commonly in traditional Chinese medicine, caused the formation of C-dots (HBCDs), which showed good dispersibility in water, broad absorption of light, and low cytotoxicity in this study. The resultant HBCDs exhibited excellent photodynamic and photothermal therapy properties along with fluorescence (FL) or PA imaging capability, which gave rise to synergistic effects in tumor treatment (Figure 7a). Furthermore, semiconducting π-conjugated polymers have been used for the preparation of functional C-dot imaging agents, as reported by Ge et al. [82]. The poly(thiophene)-based derivative PPA was synthesized via oxidative polymerization of 3-(4-(thiophen-3-yl)phenyl)propanoic acid (monomer) (Figure 7b), which was not water-dispersible (Figure 7b, inset) and showed a sheet-like shape by itself when measured by transmission electron microscopy (TEM) (Figure 7c). On the other hand, after hydrothermal carbonization, the polymer presented water-dispersible C-dots (Figure 7d, inset) as measured by TEM (Figure 7e). The resulting C-dots provided (i) multimodal imaging under visible light excitation for FL imaging and NIR irradiation for simultaneous PA imaging, and (ii) high conversion efficiency of photon energy into heat for photothermal therapy (PTT) in living mice.

**Figure 8:**
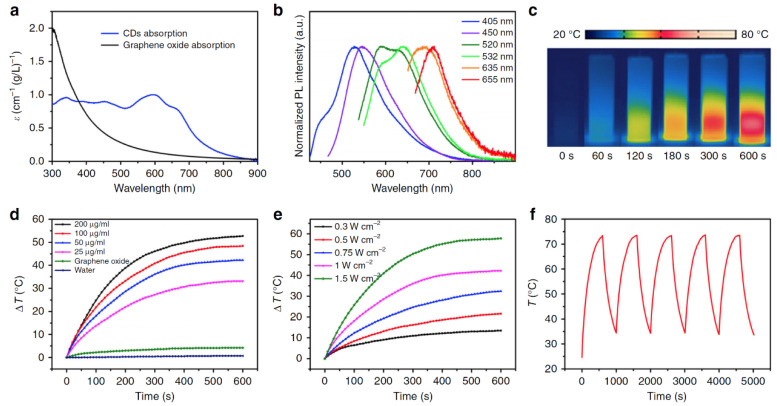
(**a**) Absorption spectra of sulfur-doped C-Dots in aqueous solution. The data from graphene oxide for comparison. (**b**) Emission spectra of the C-Dots that are excited at various wavelengths in dilute aqueous solution. (**c**) Photothermal images of the aqueous solutions of C-Dots (200 μg mL^−1^) that were captured at various times under 655-nm laser irradiation at a power density of 1 W cm^−2^. (**d**) Temperature evolutions of the C-Dots at various concentrations. The data from graphene oxide at 50 μg mL^−1^ and from pure water measured under the same conditions are shown for comparison. (**e**) Temperature evolutions of the C-Dots (50 μg mL^−1^) at various power densities. (**f**) Temperature curves of the C-Dots (200 μg mL^−1^) under five cycles of photothermal heating under 655-nm laser irradiation (1 W cm^−2^). Reproduced with permission from [83]. Copyright, Springer Nature (2020).

Elementally engineered C-dots that emit an NIR fluorescence as well as the PA signal were demonstrated. Qu et al. prepared C-dots and fine-tune their optical band gaps via a solvothermal method [83]. Herein, they used dimethyl sulfoxide (DMSO), which allowed them to dope sulfur atoms in the C-dots and to achieve the photoluminescence and photoacoustic imaging together with photothermal effect (Figure 8). Furthermore, the obtained materials could exhibit enhanced biodistribution, passive targeting, and renal clearance, which would be an essential prerequisite for advanced biomedical applications. Similarly, Li et al. demonstrated nitrogen-doped C-dot-based theragnostic agents that are capable of showing selective imaging and delivering drugs to tumors [84]. The materials were prepared from citric acid and 1,4,5,8-tetraminoanthraquinone (TAAQ) via a hydrothermal method, which caused dehydration and graphitization, forming C-dots containing α-carboxyl and amine groups on the edges of carbonized matrices. The resultant large amino acid-mimicking C-dots (LAAM TC-CQDs) showed strong absorption at 650 nm and provided near-infrared (NIR) fluorescence at 700 nm and PA imaging in the NIR region. The LAAM TC-CQDs were found to have a high binding affinity to the large neutral amino acid transporter 1 (LAT1) that can be expressed in tumors; they also show high loading efficiency of drugs as a result of the π-conjugated matrices, enabling tumor-specific imaging and chemotherapeutics.

### 3.4. Hybrid Nanocomposites

**Figure 9:**
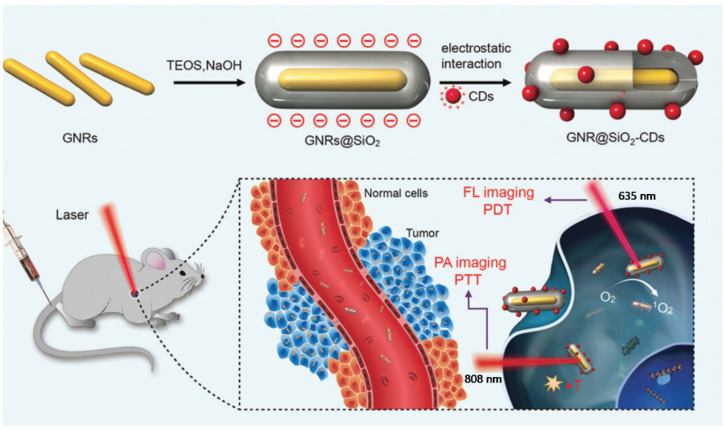
Schematic illustration of GNR@SiO_2_-CDs as a phototheragnostic agent for dual-modality FL and PA imaging-guided synergistic PDT/PTT therapy. Reprinted with permission from [91]. Copyright, The Royal Society of Chemistry (2016).

Although many carbon materials have been widely explored as a contrast agent in the PA imaging field so far, they still suffer from intrinsic issues, for instance, low absorption coefficients in the NIR region and their detectable cytotoxicity, which can limit further in vivo clinical applications [15]. To overcome these drawbacks, a variety of inorganic components have received considerable attention and demonstrated the potential to provide hybrid composites [85,86,87,88]. As a typical example, with the use of elemental gold, composite materials have been produced by forming gold nanorods (GNRs), gold nanoparticles (AuNPs), or gold layers, in conjunction with carbon materials [89,90]. Jia et al. demonstrated functional gold nanorods that are coated with silica layers and further decorated with C-dots (GNR@SiO_2_-CDs) (Figure 9). The GNRs obtained provided PA imaging and photothermal therapy while C-dots were used for FL imaging and photodynamic therapy. The silica layer between GNRs and C-dots could enhance chemical stability under physiological conditions and prevent the fluorescence quenching of C-dots. The theragnostic agents, thus, achieved FL/PA imaging-guided PDT/PTT and showed remarkable sensitivity and spatial resolution when treating cancer cells. After treatment, the composites are, in principle, discharged from the body of mice without noticeable toxicity [91].

**Figure 10:**
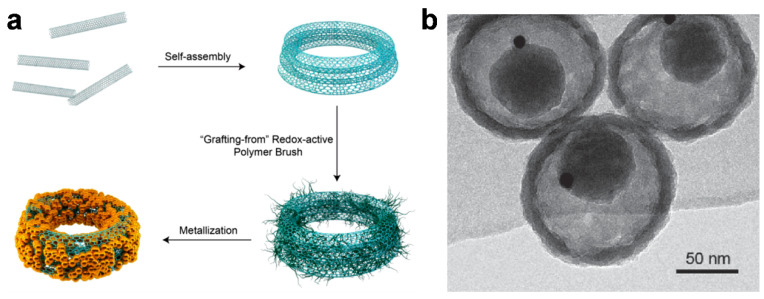
(**a**) Schematic illustration of the self-assembly of carbon nanotubes (CNTs) into a CNT ring (CNTR) and further growth of redox-active poly(4-vinylphenol) (PvPH) brushes via a surface-initiated atom-transfer radical-polymerization (SI-ATRP) method to reduce Au^3+^ to Au^0^ and coat gold nanoparticles onto the CNTR. Reprinted with permission from [92]. Copyright, American Chemical Society (2016). (**b**) Transmission electron microscopy (TEM) image of carbon-silica nanocapsules with gold nanoparticles (Au@CSN). Reprinted with permission from [93]. Copyright, John Wiley and Sons (2016).

Gold nanoparticles (AuNPs) have also been demonstrated to possess photophysical properties when incorporated in carbon-based composites. Figure 10a shows the synthetic procedure for CNT-based nano-ring composites (CNTR) having tunable diameters ranging from tens to hundreds of nanometers. Here, single-walled CNTs were efficiently aggregated and coiled to form a nanostructure via a double emulsion method in a ternary solvent system, and the surface of the materials was further coated with redox-active poly(4-vinylphenol) (PvPH) brushes via a surface-initiated atom-transfer radical-polymerization (SI-ATRP). The polymers then served as a reducing agent to induce the growth of AuNPs onto the CNTR, which played multiple roles as a probe in surface-enhanced Raman spectroscopy (SERS) and as a PA contrast agent when excited in a region of NIR irradiation. With this technique, the materials helped examine cancer cells and provided imaging-guided photothermal therapy in two tumor xenograft models [92]. AuNPs can be positioned inside as well as outside carbon composites and impart an imaging capability to the materials. Li et al. reported the preparation of carbon-based nanocapsules with AuNPs inside (Au@CSN) [93]. The inorganic nanoplatform was found to have a hollow structure consisting of interior space and a mesoporous shell, thus, AuNPs could be included inside the platform, as shown in Figure 10b. The resultant nanocapsules are biocompatible and capable of cancer theragnostics. In particular, the embedded AuNPs provided computed tomography and PA tomography as well, and the carbonaceous matrices enabled efficient photothermal therapy. Further, the encapsulation of chemodrugs in the hollow structure could cause a synergistic effect between the imaging-guided photothermal treatment and chemotherapy.

**Figure 11:**
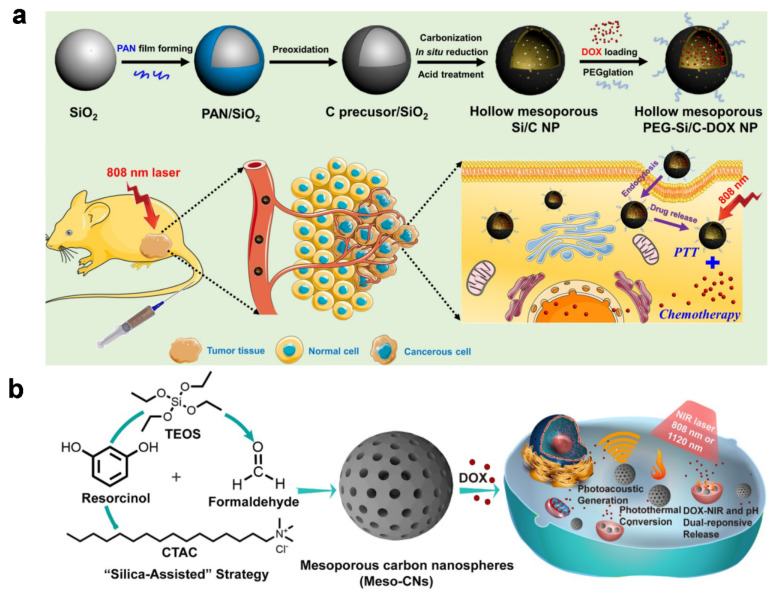
(**a**) Schematic illustration of fabrication of hollow mesoporous PEG-Si/C-DOX NP and its application for photoacoustic imaging-guided chemo-thermal therapy. Reproduced with permission from [96]. Copyright, Ivyspring International Publisher (2017). (**b**) Synthesis and application scheme for mesoporous carbon nanospheres (Meso-CNs) as photoacoustic agents and chemo-photothermal cancer therapy platforms. Reproduced with permission from [97]. Copyright, Ivyspring International Publisher (2018).

Silica (SiO_2_) is a good starting point for biocompatible materials, it has also been used extensively as a structural template for the preparation of hybrid imaging agents [94,95]. Zhang et al. reported degradable, hollow, mesoporous, composite particles [96] based on silica nanoparticles (SiO_2_ NPs) obtained from tetraethyl orthosilicate (TEOS) via hydrolyzation. APTES (3-aminopropyltriethoxysilane) is introduced onto the surface to adhere to polyacrylonitrile (PAN) as a carbon source; this functions via hydrogen bonds between the primary amine in APTES and the nitrile in PAN, giving polymeric nanoparticles (PAN/SiO_2_). Subsequent thermal carbonization results in the formation of carbon-coated layers on the Si core and further acid treatment forms hydrophilic functionalities such as carboxyl or hydroxyl groups. The resultant particles (Si/C NPs) are then loaded with doxorubicin (DOX) and the outer surface of the materials is further functionalized with polyethylene glycol (PEG) to form the desired composite particles (PEG-Si/C-DOX NPs) that are capable of PA imaging-guided photothermal treatment or chemotherapy. The particles accumulate at the desired location because of their size, providing efficient cancer treatment upon irradiation with NIR light; they also biodegrade under physiological conditions, making them a multifunctional, biocompatible agent for cancer theragnostics (Figure 11a). Figure 11b shows spherical carbon composites that have mesopores (diameter ~2.5 nm), as reported by Zhou et al. [97]. In the synthesis, TEOS and charged surfactants (hexadecyl trimethylammonium chloride; CTCA) are used to form the mesoporous silica-based framework together with resorcinol and formaldehyde under basic conditions. Calcination then produces mesoporous carbon nanospheres (Meso-CNs) that are able to absorb a wide spectrum of light (i.e., 300–1400 nm) that induces more efficient PA imaging and photothermal conversion in comparison with SWCNT or gold nanorods. Furthermore, mesopores can substantially enhance the properties of composite materials [98]. Their size, volume, and high specific surface area allow them to be loaded with DOX for chemotherapy at a high capacity (35 wt.%) and to produce a triggered release of the drug molecules in response to pH or NIR light. In addition to PAN or the aromatic small molecules shown, many organic materials have the capability of providing carbonaceous matrices. For example, microporous organic polymers generally show a very high char yield when carbonized [99,100,101,102], a strong requirement for the formation of matrices during carbonization. Different types of other materials with fibrous or hierarchical structures or from natural sources also facilitate the synthesis of matrices [103,104,105,106,107].

**Figure 12:**
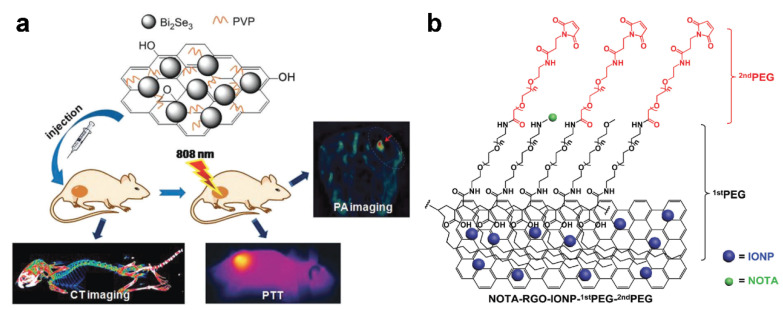
(**a**) Schematic diagram of GO/Bi_2_Se_3_/PVP nanocomposites for CT/PA imaging and PTT. Reprinted with permission from [86]. Copyright, The Royal Society of Chemistry (2017). (**b**) Schematic illustration of the structure of the NOTA-RGO-IONP-^1st^PEG-^2nd^PEG nanocomposite. Reprinted with permission from [85]. Copyright, The Royal Society of Chemistry (2016).

Graphene is one of the carbon allotropes and has a π-conjugated, two-dimensional structure. As a result of its large surface area and unique electronic properties, the layered material has been widely used for the formation of carbon-based composites. Zhang et al. deposited Bi_2_Se_3_ nanoparticles onto a graphene oxide (GO) layer in the presence of poly(vinylpyrrolidone) (PVP) and formed the GO/Bi_2_Se_3_/PVP nanocomposites via a solvothermal method [86], as shown in Figure 12a. The elemental bismuth has good biocompatibility despite its heavy metal nature and can provide high computed tomography (CT) contrast because of its large X-ray attenuation coefficient (Bi, 5.74 cm^2^ kg^–1^ at 100 eV), overcoming the disadvantages of widely used iodine-based agents, including renal toxicity and a short imaging window. At the same time, the nano-sized GO not only acts as a structural support but also enables PA imaging and photothermal therapy using NIR irradiation at 808 nm. In general, CT has the advantages of deep tissue penetration, high resolution, and facile construction of three-dimensional (3D) images, while PA imaging offers high contrast in soft tissues because of good spatial resolution and high sensitivity. Therefore, the nanomaterial platforms can combine both these advantages to better locate target cells and accurately guide PTT.

Reduced graphene oxides (RGOs) have also provided a versatile platform for nanocomposites. For example, Xu et al. developed functional RGO-based composites that are capable of multimodal imaging and do not interfere with blood circulation [85]. The multicomponent materials were formed using RGO sheets that were decorated with iron oxide nanoparticles (IONPs) and also encapsulated with polyethylene glycols (PEGs), which resulted in the cocktail materials, RGO-IONP-^1st^PEG-^2nd^PEG, as shown in Figure 12b. Taking advantage of strong NIR absorption and superparamagnetism, the designed agents could achieve positron emission tomography (PET) imaging and magnetic resonance imaging (MRI) along with PA imaging. Furthermore, the overall size of the materials allows them to be selectively accumulated in tumors as a result of the enhanced permeability and retention (EPR) effect; this suggests the value of nanoconjugation on RGOs and points to a rational design process for tumor-targeting theragnostic agents.

**Figure 13:**
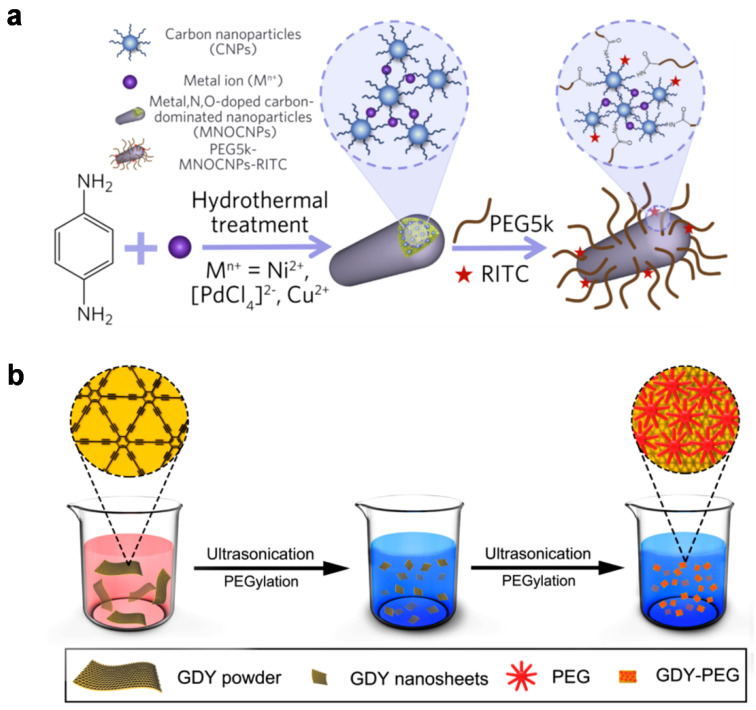
(**a**) Schematic illustrations of hydrothermal preparation and further modification of metal, N, O-doped carbon nanoparticles (MNOCNPs). Reprinted with permission from [108]. Copyright, Elsevier (2019). (**b**) Synthesis of the fabrication process of the PEG-functionalized graphdiyne material (GDY-PEG). Reprinted with permission from [118]. Copyright, American Chemical Society (2017).

A variety of inorganic additives and novel carbon allotropes have also attracted attention since these hybrids are able to show unprecedented yet comprehensively validated outcomes of having the desired photophysical properties for imaging. As an example, Bao et al. developed metal-doped carbonaceous nanocomposites via a single-step hydrothermal method [108]. As shown in Figure 13a, the particles were formed predominantly from *p*-phenylenediamine as a carbon source in the presence of trace metal ions such as Ni, Pd, or Cu. This results in the formation of metal-, N-, or O-doped carbon-based wire-like nanocomposites (MNOCNPs) that exhibit high NIR absorption, photothermal properties, and biocompatibility while also being conjugated with polyethylene glycol (PEG) and rhodamine B isothiocyanate (RITC) for fluorescence (FL) imaging. Taking advantage of their surface properties and overall size (~40 nm), the materials show physiological features such as the endoplasmic reticulum and tumor accumulation without causing liver damage. Therefore, the agent materials could be used for PA/FL/thermal imaging-guided cancer treatment; they hold the promise of a nucleolar delivery system triggered by NIR irradiation that addresses lysosomal entrapment and liver damage. Although we only present here the incorporation of several specific metal ions, other metal ions (including lanthanide (III) ions) that have unique physical properties, for example, long-lasting luminescence or paramagnetic properties, have the potential for further functionalization of carbon matrices [109,110,111,112,113,114,115]. 

Two-dimensional materials have received great interest and broadened their scope of application in the fields of energy storage, energy conversion, sensing, and catalysis [116,117]. Recently, Li et al. reported nanotransducers that can provide simultaneous PA imaging and photothermal therapy using graphdiyne (GDY), a two-dimensional carbon-based network comprised of benzene rings and acetylene linkages [118]. This novel sp- and sp^2^-hybridized carbon network was first demonstrated in 2010 [119] and has now shown unique properties such as uniform pore size, strong NIR absorption, and controllable electronic properties. The GDY sheets can be encapsulated with polyethylene glycol (PEG) via intermolecular electrostatic interactions, which results in the nanocomposite GDY-PEG (Figure 13b); the materials show biocompatibility and efficient photothermal ablation of cancer cells in response to laser irradiation at 808 nm. Similarly, other 2D materials such as metal chalcogenides or MXenes that have recently received attention are thought to provide the platform for formulating theragnostic agents similar to GDY or RGO sheets, which then may have synergistic or enhanced photophysical properties [120,121,122].

**Figure 14:**
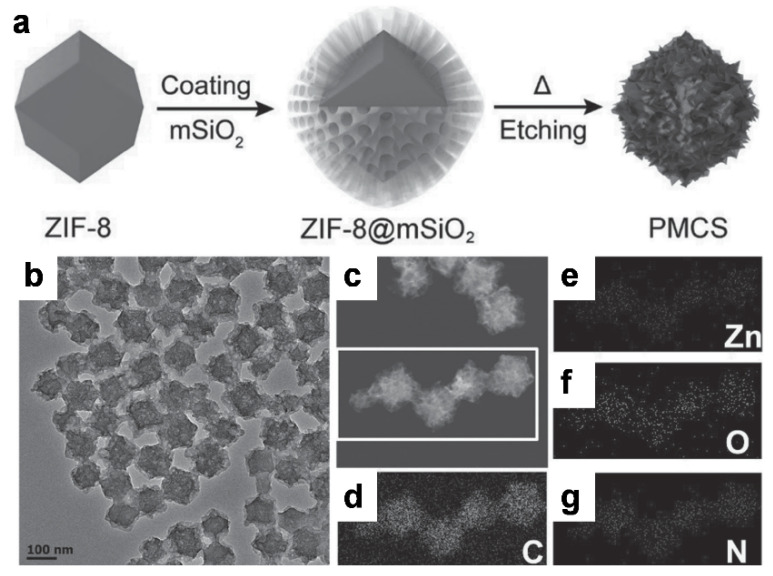
Synthesis and characterization data for mesoporous carbon nanospheres containing porphyrin-like metal centers (PMCS). (**a**) Scheme for PMCS synthesis; (**b**) TEM and (**c**) STEM images, scale bar is 100 nm. Element mapping for (**d**) carbon, (**e**) zinc, (**f**) oxygen, and (**g**) nitrogen. Reproduced with permission from [123]. Copyright, John Wiley and Sons (2016).

Metal-organic frameworks (MOFs) have provided a platform for the formation of carbon nanocomposites which are used in bio-imaging and phototherapy. Wang et al. demonstrated the formation of spherical carbon-based nanocomposites using the zeolitic imidazolate framework-8 (ZIF-8) [123]. The synthetic precursor was encapsulated by a mesoporous silica shell (SiO_2_) followed by pyrolysis at 800 °C and etching with sodium hydroxide; these resulted in the formation of monodisperse, metal-doped carbon nanospheres (PMCS) (Figure 14). The resulting materials contained meso-sized pores and porphyrin-like zinc centers (confirmed by elemental mapping) that played a role as photosensitizer in the carbon matrix; these were further coated with polyethylene glycol-vitamin E (PEG-VE) for biocompatibility. As designed, the PEGylated PMCS showed good stability and high photothermal conversion under 808 nm irradiation, and further enabled PA imaging and provided photodynamic properties for cancer treatment.

## 4. Concluding Remarks

Photo-acoustic imaging is a new class of biomedical methods that can provide multi-contrast images with deep penetration depth and high spatial resolution much sought after in clinical applications. The hybrid images obtained from PAI are generally derived from a combination of optical and acoustic signals; the imaging methodology can be further classified in detail as PA microscopy or PA computer tomography, depending on the configuration of the entire system. Together with suitably developed instrumentation, exogenous agents can play a remarkable role not only in increasing the contrast and sensitivity of PA images but also in giving rise to other desired functions such as therapeutic effects. Among many candidate materials, carbon-based agents have been widely researched for these bio-imaging applications in the form of nanotubes, nanohorns, nanodots, or nanosheets. They have been embedded in a variety of matrices to provide photoacoustic signals, as extensively summarized in this review. We anticipate that the imaging techniques and the contrast agents described will be further developed into more advanced materials and to monitor or manipulate them using the PA signals generated from the materials [124,125]. Therefore, on the one hand, the agent materials can be incorporated into biocompatible polymers such as hydrogels or bio-based thermosets, to enhance sensing ability as a signal transducer for PA signals under physiological conditions [123,124,125,126,127,128,129,130,131,132]. On the other hand, they would provide spatiotemporal control of stimuli-responsive materials that exhibit auto-inductive or reversible responses [133,134,135,136,137,138] or would be used to analyze interior structures when embedded in complex materials containing biomimetic or hierarchical structures [104,117,139,140,141]. Furthermore, the functional carbon-based materials would open up the possibilities for the development of theragnostic biomedical devices, e.g., smart vascular scaffolds or self-powered healthcare systems that can sensitively monitor diverse bio-signals on the basis of PA signals and provide an early, relevant treatment when needed.

## Data Availability

Not applicable.
